# Inflammatory-induced swelling after total knee arthroplasty: Obesity and preoperative joint pain as key predictors

**DOI:** 10.1186/s42836-025-00344-9

**Published:** 2025-11-13

**Authors:** Lin Yang, Yi-fang Zhan, Zan-jing Zhai, Shi-Zhen Zhang, Cai-feng Wang, Qun Li, Bei-Ying Wu, Wei-Wei Bian, Hui-Wu Li, Hong Ruan

**Affiliations:** 1 Department of Nursing, Shanghai Ninth People’s Hospital, Shanghai JiaoTong University School of Medicine, Shanghai, 200011 China; 2 School of Nursing, Shanghai JiaoTong University, Shanghai, 200025 China; 3 Department of Orthopaedics, Shanghai Ninth People’s Hospital, Shanghai JiaoTong University School of Medicine, Shanghai, 200011 China; 4 Rehabilitation Department, Shanghai Ninth People’s Hospital, Shanghai JiaoTong University School of Medicine, Shanghai, 200011 China; 5 Shanghai Nursing Association, Shanghai, 200040 China

**Keywords:** Total knee arthroplasty, Swelling, Inflammatory response, Body Mass Index, Pain

## Abstract

**Objective:**

Limited research exists on factors influencing early postoperative swelling after total knee arthroplasty (TKA). This prospective observational cohort study aims to analyze patterns and predictors of early postoperative inflammatory swelling following TKA, informing high-risk screening and interventions.

**Methods:**

This study measured 110 TKA patients’ (76.4% female, mean age 69.51 ± 7.46 years) lower limb circumference (10 cm/5 cm above patella, patella, calf, ankle) preoperatively and postoperatively 1–5 days. Variables of interest included body mass index (BMI), preoperative joint pain, postoperative joint and thigh pain, cryotherapy duration, and inflammatory biomarkers.

**Results:**

Swelling peaked proximally at 5 cm above the patella on day 3 (4.16 ± 2.90 cm). Multivariable regression analysis identified BMI as a core predictor of lower limb swelling (*P* < 0.05), with a 1-unit increase in BMI corresponding to a 0.18–0.24 cm increase in proximal leg circumference. Pain timing was site-specific: swelling 10 cm above the patella was associated with preoperative joint pain on touch (β = 0.184, *P* = 0.052), calf swelling with postoperative joint pain at rest (β = 0.213, *P* = 0.022), and ankle swelling with postoperative thigh pain at rest (β = 0.228, *P* = 0.017). Other factors and inflammatory markers were not significantly associated with swelling (*P* > 0.05). However, preoperative joint pain during activity correlated with higher white blood cell (WBC) counts (*P* = 0.012), and postoperative thigh resting pain with elevated C-reactive protein (CRP) levels (*P* = 0.035).

**Conclusion:**

Early postoperative proximal swelling following TKA warrants attention, as chronic pro-inflammatory states in obese patients and the progression of knee osteoarthritis lead to elevated inflammatory levels, exacerbating postoperative swelling. Focus should be placed on high-risk patients with obesity and preoperative joint pain on touch.

**Trial registration:**

ChiCTR2500098167.

Video Abstract

**Supplementary Information:**

The online version contains supplementary material available at 10.1186/s42836-025-00344-9.

## Introduction

Total Knee Arthroplasty (TKA) is an effective treatment for end-stage knee osteoarthritis (KOA), significantly improving joint function and quality of life. However, it is reported that up to 90% of patients experience swelling after TKA [1]. Joint-related muscle inhibition leads to quadriceps activation difficulties and a decrease in extensor strength, resulting in delayed rehabilitation [2, 3]. Swelling limits joint flexion and restricts rehabilitation exercises, impacting early postoperative joint range of motion and hindering the recovery process. Within 90 days post-surgery, 1.4% to 17.5% of patients seek emergency treatment for swelling [4], placing a burden on the healthcare system. The incidence of knee joint swelling at 6 months post-surgery is 40.0%, while calf swelling occurs in 23.9%. One year post-TKA, the incidence of knee joint swelling is 26.9%, with calf swelling at 19.1% [5]. Therefore, controlling postoperative swelling after TKA is crucial.

Postoperative swelling following TKA is not caused by a single factor; however, existing intervention studies often overlooked this point, which leads to suboptimal outcomes [6, 7]. Based on literature and consensus among multidisciplinary experts, four potential mechanisms related to postoperative swelling after TKA were identified: inflammatory response, venous return insufficiency, joint hematoma, and muscle injury and healing [8]. Among these, the inflammatory response is an important factor, as TKA involves bone tissue trauma and ischemia–reperfusion injury from the use of a tourniquet, which can trigger inflammatory responses. Additionally, the stress stimulation caused by rehabilitation activities may prolong this inflammatory response, which can be quantified by inflammatory biomarkers, such as white blood cell (WBC) count and C-reactive Protein (CRP) level. However, the correlation between the inflammatory response and the degree of swelling remains unclear. Some studies have proposed factors that influence postoperative swelling and analyzed their potential links to inflammatory responses, such as Body Mass Index(BMI), history of diabetes, and perioperative pain [9], though the associations are not well-defined. Moreover, while cryotherapy is widely used in clinical settings to reduce swelling, the heterogeneity in swelling control effects remains controversial, limiting the optimization of personalized intervention strategies [10]. Therefore, this study employs a prospective observational cohort study design to describe the distribution patterns of lower limb swelling across various anatomical sites, investigate the potential effects of individual factors and interventions on inflammation-induced swelling, examine the relationship between inflammatory markers and the severity of swelling, and use multivariate regression models to identify high-risk populations.

## Participants and methods

### Participants

Convenient sampling was used to select patients who were hospitalized in the orthopedic department of a tertiary hospital in Shanghai from November 2024 to April 2025. Inclusion criteria: (1) diagnosed with KOA and meeting the indications for TKA; (2) aged ≥ 18 years; (3) scheduled to undergo unilateral TKA; (4) able to understand patient information and sign the informed consent form. Exclusion criteria: (1) patients with severe medical conditions; (2) patients with cognitive impairments who are unable to effectively communicate their intentions or cooperate with the trial.

## Methods

### Study design

This study used a prospective observational cohort design.

#### Standardized surgical and perioperative protocol

All TKA procedures followed the standardized clinical pathway, which is a well-established practice in our setting. A pneumatic tourniquet was routinely used in all cases. The tourniquet pressure was set at 35 kPa. It was inflated after the completion of the osteotomy and before prosthesis installation, and deflated after the prosthesis was cemented and the cement had hardened. Based on prior data from our center (*n* = 178), the tourniquet time under this protocol exhibited minimal inter-individual variation (mean: 9.5 ± 1.6 min). At the end of the surgery, before closing the joint cavity, directly inject 1 g of tranexamic acid solution into the joint cavity, and then close the incision.

#### Data collection

The study was approved by the Medical Ethics Committee of the Ninth People’s Hospital affiliated to Shanghai Jiaotong University School of Medicine, Shanghai, China (SH9H-2023-TK323-1), and registered as a clinical trial (ChiCTR2500098167). Data collection included: (1) Basic Information: Data were extracted from the electronic medical records of the patients, including age, gender, BMI, history of diabetes, and surgical site (left/right lower limb). (2) Lower Limb Swelling: Measurements were taken by a well-trained nurse using a soft tape measure with an accuracy of 0.1 cm on the day before surgery and on postoperative days 1 to 5. The measurements included the circumference of 10 cm and 5 cm above the patella, patella, calf, and ankle. The patient was positioned supine, with no vigorous joint movements before or after measurements. The difference in leg circumference between postoperative and preoperative measurements was used as the swelling index. (3) Pain: Pain was first assessed using a Visual Analog Scale (VAS) (1–10 points) by trained nursing staff at the same time points as the swelling measurements. For patients with pain over 0 points, the pain characteristics, timing (activity/rest/touch), and pattern (sudden onset/intermittent/persistent) were further inquired about. (4) Postoperative Cryotherapy Duration (min/d): The duration was recorded based on patient or family member reports. (5) Blood Test Indicators: Preoperative and 24-h postoperative inflammatory markers were obtained from the hospital laboratory information system, including WBC count (10⁹/L) and CRP (mg/L). Postoperative data were strictly limited to the first postoperative 24-h follow-up results.

### Statistical analysis

All data were independently entered into an electronic database by two researchers, with quality control performed through logical checks (e.g., validity of value ranges) and cross-validation (e.g., discrepancies > 5% triggering review of original records). Extreme values (e.g., dimension difference > 10 cm) were confirmed through clinical records and imaging, and a complete and clean dataset was created for analysis. SPSS 26.0 software was used for statistical analysis. Continuous variables were expressed as mean ± standard deviation, and categorical variables as frequency (percentage). Swelling changes at different anatomical sites were plotted using Microsoft Excel. Pearson correlation analysis was used to assess the relationship between various variables and the degree of swelling at different anatomical sites. A multiple linear regression analysis (backward method) was conducted to explore the impact of continuous variables on postoperative swelling at various lower limb sites. Model significance was assessed using the F-test (α = 0.05). The effect of variables was quantified using unstandardized coefficients (B values) and their 95% confidence intervals (95% CI), while standardized coefficients (β) were used to compare the relative strength of variable effects. Multicollinearity was diagnosed using the variance inflation factor (VIF, with VIF < 10 being acceptable), and the robustness of the results was validated using the Bootstrap method (1000 resamples).

## Results

### General information

A total of 110 TKA patients were included in this study, with ages ranging from 41 to 85 years (mean age: 69.51 ± 7.46 years). The BMI range was 15.50–38.97 kg/m^2^ (mean BMI: 25.88 ± 3.92 kg/m^2^). Preoperative joint pain score was 6.11 ± 1.76, with WBC count ranging from (2.45–10.64) × 10⁹/L (mean: (6.11 ± 1.60) × 10⁹/L) and CRP ranging from 0 to 137.00 mg/L (mean: 4.50 ± 14.40 mg/L). Postoperative joint pain score was 6.45 ± 1.98, thigh pain score was 5.53 ± 2.61, and the average duration of cryotherapy was 34.95 ± 14.60 min/d (range: 0–75 min/d). Postoperative WBC count increased to (10.95 ± 2.61) × 10⁹/L, and CRP levels rose to 26.15 ± 25.12 mg/L. Detailed information is presented in Table 1.

**Table 1:** Postoperative lower limb swelling across subgroups (Mean ± SD)

Variable	Subgroup	*n*	%	Lower limb sites				
				**10 cm above patella**	**5 cm above patella**	**Patella**	**Calf**	**Ankle**
**Age (years)**								
	< 65	23	20.9	4.16 ± 3.70	4.33 ± 3.33	4.18 ± 3.16	1.70 ± 3.70	0.50 ± 2.92
	65–75	64	58.2	3.40 ± 2.74	3.71 ± 2.85	3.46 ± 3.20	1.96 ± 3.14	−0.23 ± 1.38
	> 75	23	20.9	3.70 ± 3.60	3.34 ± 2.04	3.61 ± 2.07	1.87 ± 2.81	0.54 ± 1.18
**Gender**								
	Male	26	23.6	4.39 ± 3.37	4.24 ± 2.78	4.17 ± 2.74	2.24 ± 3.12	0.13 ± 1.58
	Female	84	76.4	3.38 ± 3.04	3.61 ± 2.82	3.48 ± 3.05	1.78 ± 3.20	0.06 ± 1.87
**Surgical Site**								
	Left	51	46.4	3.55 ± 3.41	3.84 ± 2.75	3.52 ± 2.54	2.16 ± 3.63	0.13 ± 2.07
	Right	59	53.6	3.67 ± 2.90	3.69 ± 2.88	3.75 ± 3.33	1.65 ± 2.73	0.04 ± 1.54
**History of diabetes**								
	Present	26	23.6	2.91 ± 2.80	3.28 ± 2.12	3.27 ± 2.03	1.71 ± 2.42	−0.29 ± 1.70
	Absent	84	76.4	3.84 ± 3.21	3.91 ± 2.98	3.76 ± 3.22	1.94 ± 3.38	0.19 ± 1.82
**BMI group**								
	Underweight	1	0.9	−0.60 ± 0.00	2.00 ± 0.00	1.90 ± 0.00	2.60 ± 0.00	1.00 ± 0.00
	Normal	35	31.8	3.39 ± 2.24	3.45 ± 2.12	3.17 ± 2.79	1.17 ± 2.26	−0.10 ± 1.31
	Overweight	41	37.3	3.21 ± 3.06	3.22 ± 2.72	3.52 ± 2.95	1.54 ± 3.45	−0.26 ± 1.47
	Obese	33	30.0	4.49 ± 3.84	4.81 ± 3.34	4.34 ± 3.19	3.06 ± 3.44	0.66 ± 2.44
**Pre-op joint pain during activity**								
	Present	102	92.7	3.70 ± 3.17	3.86 ± 2.86	3.73 ± 3.03	1.91 ± 3.16	0.10 ± 1.76
	Absent	8	7.3	2.54 ± 2.43	2.54 ± 1.80	2.53 ± 2.10	1.65 ± 3.64	−0.13 ± 2.40
**Pre-op joint pain at rest**								
	Present	33	30.0	3.26 ± 2.85	3.13 ± 2.58	3.32 ± 3.27	1.84 ± 2.74	0.22 ± 1.27
	Absent	77	70.0	3.77 ± 3.25	4.03 ± 2.88	3.78 ± 2.85	1.91 ± 3.36	0.02 ± 1.99
**Pre-op joint pain on touch**								
	Present	14	12.7	5.26 ± 3.08	4.99 ± 3.56	4.86 ± 3.20	2.61 ± 3.43	−0.03 ± 1.72
	Absent	96	87.3	3.38 ± 3.08	3.58 ± 2.66	3.46 ± 2.92	1.78 ± 3.14	0.10 ± 1.82
**Pre-op joint pain with sudden onset**								
	Present	58	52.7	3.45 ± 2.54	3.83 ± 1.88	3.75 ± 2.66	2.19 ± 2.79	0.26 ± 1.79
	Absent	52	47.3	3.80 ± 3.70	3.68 ± 3.59	3.52 ± 3.32	1.55 ± 3.56	−0.12 ± 1.81
**Pre-op joint intermittent pain**								
	Present	14	12.7	5.26 ± 3.08	4.99 ± 3.56	4.86 ± 3.20	2.61 ± 3.43	−0.03 ± 1.72
	Absent	96	87.3	3.38 ± 3.08	3.58 ± 2.66	3.46 ± 2.92	1.78 ± 3.14	0.10 ± 1.82
**Pre-op joint persistent pain**								
	Present	58	52.7	3.45 ± 2.54	3.83 ± 1.88	3.75 ± 2.66	2.19 ± 2.79	0.26 ± 1.79
	Absent	52	47.3	3.80 ± 3.70	3.68 ± 3.59	3.52 ± 3.32	1.55 ± 3.56	−0.12 ± 1.81
**Post-op joint pain during activity**								
	Present	23	20.9	3.22 ± 3.85	3.64 ± 4.07	3.49 ± 3.75	1.50 ± 4.46	−0.03 ± 2.14
	Absent	87	79.1	3.72 ± 2.93	3.79 ± 2.40	3.68 ± 2.76	1.99 ± 2.76	0.11 ± 1.71
**Post-op joint pain at rest**								
	Present	38	34.5	3.90 ± 3.38	3.57 ± 3.00	3.77 ± 3.25	1.83 ± 2.63	−0.06 ± 1.48
	Absent	72	65.5	3.47 ± 3.01	3.86 ± 2.72	3.58 ± 2.85	1.92 ± 3.44	0.15 ± 1.95
**Post-op joint pain on touch**								
	Present	100	90.9	3.69 ± 2.93	3.81 ± 2.63	3.70 ± 2.88	2.01 ± 3.07	0.15 ± 1.77
	Absent	10	9.1	2.95 ± 4.89	3.26 ± 4.37	3.10 ± 3.94	0.63 ± 4.02	−0.64 ± 2.03
**Post-op joint pain with sudden onset**								
	Present	53	48.2	3.62 ± 3.00	3.73 ± 2.38	3.26 ± 2.07	2.61 ± 2.63	0.44 ± 1.71
	Absent	57	51.8	3.61 ± 3.28	3.79 ± 3.18	3.99 ± 3.61	1.21 ± 3.50	−0.26 ± 1.83
**Post-op joint intermittent pain**								
	Present	34	30.9	4.36 ± 3.46	4.31 ± 3.19	3.85 ± 3.21	2.11 ± 3.51	−0.13 ± 2.02
	Absent	76	69.1	3.29 ± 2.94	3.51 ± 2.61	3.55 ± 2.88	1.79 ± 3.03	0.18 ± 1.70
**Post-op joint persistent pain**								
	Present	38	34.5	3.28 ± 2.56	3.88 ± 2.07	3.87 ± 3.13	1.95 ± 2.53	0.11 ± 1.15
	Absent	72	65.5	3.80 ± 3.40	3.70 ± 3.14	3.52 ± 2.91	1.86 ± 3.48	0.06 ± 2.07
**Post-op thigh pain during activity**								
	Present	82	74.5	3.59 ± 2.77	3.86 ± 2.44	3.70 ± 2.76	2.09 ± 2.81	0.05 ± 1.70
	Absent	28	25.5	3.70 ± 4.07	3.48 ± 3.73	3.49 ± 3.60	1.29 ± 4.06	0.15 ± 2.09
**Post-op thigh pain at rest**								
	Present	49	44.5	3.37 ± 2.53	3.67 ± 2.23	3.47 ± 2.67	2.47 ± 2.91	0.53 ± 1.69
	Absent	61	55.5	3.82 ± 3.55	3.83 ± 3.22	3.78 ± 3.22	1.42 ± 3.33	−0.29 ± 1.81
**Post-op thigh pain on touch**								
	Present	27	24.5	3.91 ± 3.09	3.65 ± 2.75	3.68 ± 2.92	2.04 ± 3.21	−0.37 ± 2.07
	Absent	83	75.5	3.52 ± 3.16	3.80 ± 2.84	3.63 ± 3.01	1.84 ± 3.18	0.23 ± 1.69
**Post-op thigh pain with sudden onset**								
	Present	40	36.4	3.52 ± 2.90	4.01 ± 2.29	3.47 ± 2.45	1.98 ± 2.45	−0.03 ± 1.08
	Absent	70	63.6	3.68 ± 3.28	3.62 ± 3.07	3.74 ± 3.25	1.84 ± 3.54	0.14 ± 2.11
**Postop thigh intermittent pain**								
	Present	19	17.3	4.39 ± 2.56	4.83 ± 2.82	4.51 ± 2.51	1.58 ± 3.38	0.16 ± 2.93
	Absent	91	82.7	3.46 ± 3.23	3.54 ± 2.77	3.46 ± 3.05	1.95 ± 3.15	0.06 ± 1.49
**Post-op thigh persistent pain**								
	Present	59	53.6	3.38 ± 2.63	3.45 ± 2.17	3.28 ± 2.61	2.21 ± 2.90	0.34 ± 1.91
	Absent	51	46.4	3.90 ± 3.64	4.12 ± 3.39	4.06 ± 3.33	1.51 ± 3.46	−0.22 ± 1.63
**Cryotherapy duration (mins/day)**								
	< 30	13	11.8	3.24 ± 2.35	3.32 ± 2.15	2.47 ± 2.43	1.79 ± 2.38	−0.12 ± 1.29
	30–59	86	78.2	3.72 ± 3.23	3.87 ± 2.90	3.70 ± 3.05	1.94 ± 3.23	0.01 ± 1.87
	≥ 60	11	10.0	3.24 ± 3.30	3.45 ± 2.94	4.55 ± 2.74	1.58 ± 3.74	0.84 ± 1.68
**Post-op WBC**								
	Normal	35	31.8	3.12 ± 2.75	3.47 ± 2.63	3.79 ± 3.29	2.81 ± 3.53	0.31 ± 1.50
	Elevated	75	68.2	3.85 ± 3.29	3.90 ± 2.90	3.57 ± 2.84	1.46 ± 2.92	−0.03 ± 1.92
**Post-op CRP**								
	Normal	25	22.7	3.40 ± 3.45	3.75 ± 2.78	3.35 ± 2.86	1.58 ± 2.28	0.15 ± 0.93
	Elevated	85	77.3	3.73 ± 3.05	3.76 ± 2.83	3.68 ± 3.05	1.98 ± 3.40	0.06 ± 1.99
**Total**		110	100	3.62 ± 3.13	3.76 ± 2.81	3.64 ± 2.98	1.89 ± 3.17	0.08 ± 1.80

Pre-op: Preoperative; Post-op: Postoperative; WBC: White Blood Cell Count; CRP: C-Reactive Protein

### Distribution patterns of lower limb swelling after TKA

**Fig. 1:**
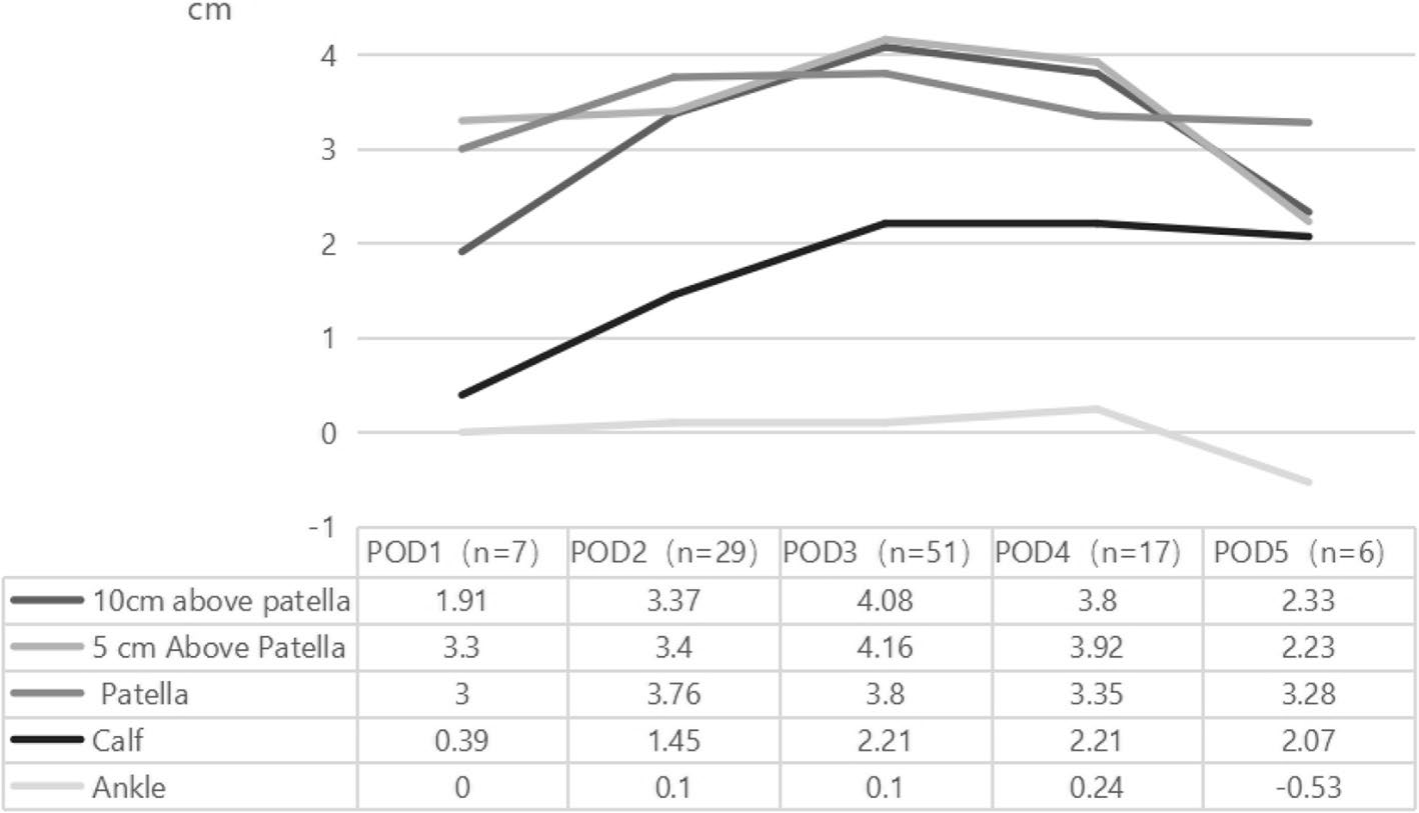
Changes in swelling at anatomical sites of the lower limb after TKA

There are differences in the degree of swelling at different anatomical sites postoperatively (Table 2). The greatest swelling occurred at the 5 cm above the patella (3.76 ± 2.81 cm), followed by the patella, 10 cm above the patella, calf, and ankle. The dynamic changes in swelling are shown in Fig. 1. Analysis of the most severe swelling at each anatomical site on different postoperative days revealed that swelling at the 5 cm above the patella site was the most severe on postoperative days 1, 3, and 4, while swelling at the patella was most severe on postoperative days 2 and 5. In terms of peak swelling at each anatomical site, ankle swelling peaked on postoperative day 4, while all other sites reached their peak on postoperative day 3. The greatest swelling was observed at the 5 cm above the patella site, with a peak of 4.16 ± 2.90 cm.

**Table 2:** Lower limb swelling after TKA (cm)

Anatomical Site	Range Difference	Median	Minimum	Maximum	P25, P75
10 cm above patella	18.30	3	−5.70	12.60	1.975, 5.425
5 cm above patella	15.60	3.6	−3.50	12.10	2, 5
Patella	19.10	3.3	−3.60	15.50	1.8, 5
Calf	19.60	1.75	−6.10	13.50	−0.05, 3.325
Ankle	13.50	0.1	−4.00	9.50	−0.925, 0.925

### Comparison of lower limb swelling levels in different subgroups of TKA patients

The Mann–Whitney U test was used to analyze the differences in postoperative swelling levels between subgroups. It was found that patients who had preoperative joint pain on touch exhibited significantly more swelling at the 10 cm above the patella site (*P* < 0.05). Patients who had pain at rest in the joint or thigh postoperatively experienced more significant swelling in the calf and ankle (*P* < 0.05), as shown in Table 3. To further investigate the potential causes of swelling in relation to pain characteristics, inflammatory markers were also analyzed. Preoperative joint pain during activity was associated with significantly elevated WBC counts (9.79 vs 11.04, *P* = 0.012). Postoperative thigh pain at rest was associated with a significant increase in CRP levels (21.3 vs 32.2, *P* = 0.035), as shown in Table 4. No significant differences were found in swelling levels between other groups (*P* > 0.05). Analysis of the mean swelling levels across groups (Table 1) revealed that male patients had higher swelling levels than female patients. The subgroup of patients under 65 years old had greater swelling at the 5 cm above the patella, 10 cm above the patella, and the patella sites compared to other age groups. Patients without a history of diabetes and those who were obese (BMI ≥ 30 kg/m^2^) experienced greater swelling at all sites compared to other groups. In the postoperative inflammatory indicator subgroup, the group with elevated WBC counts showed higher swelling at the 10 cm above the patella and 5 cm above the patella, but lower swelling at other sites. The group with elevated CRP levels showed higher swelling at all sites except the ankle.

**Table 3:** Mann–Whitney U test results for postoperative swelling and pain characteristics (significant results)

Pain characteristics	Anatomical site	subgroup	*n*	Mean rank	Mann–Whitney U value	Z value	*P*-value
**Pre-op joint pain on touch**	10 cm above patella	Present	14	72.96	427.500	−2.194	0.028*
		Absent	96	52.95			
**Post-op joint pain at rest**	Calf	Present	53	64.82	1016.500	−2.956	0.003*
		Absent	57	46.83			
	Ankle	Present	53	61.74	1180.000	−1.979	0.048*
		Absent	57	49.70			
**Post-op thigh pain at rest**	Calf	Present	49	62.50	1151.500	−2.063	0.039*
		Absent	61	49.88			
	Ankle	Present	49	62.98	1128.000	−2.206	0.027*
		Absent	61	49.49			

^*^ indicates statistical significance

**Table 4:** Comparison of inflammatory markers by pain timing (significant results)

Variable	Group	*n*	Mean ± SD	*t* Value	*P*-Value	95% Confidence Interval
**Pre-op joint pain during activity**						
**CRP (mg/L)**	Present	102	26.2 ± 25.8	−0.054	0.957	−18.87, 17.86
	Absent	8	25.7 ± 14.3			
**WBC (× 10⁹/L)**	Present	102	11.04 ± 2.67	−2.812	0.012*	−2.19, −0.31
	Absent	8	9.79 ± 1.01			
**Post-op thigh pain at rest**						
**CRP (mg/L)**	Present	49	32.2 ± 32.3	−2.153	0.035*	−20.95, −0.79
	Absent	61	21.3 ± 16.1			
**WBC (× 10⁹/L)**	Present	49	11.17 ± 2.88	−0.788	0.433	−1.39, 0.62
	Absent	61	10.78 ± 2.37			

^*^indicates statistical significance

### Correlation analysis of postoperative swelling and various variables

Spearman correlation analysis revealed a significant positive correlation between BMI and swelling at the 5 cm above the patella (r = 0.208, *P* = 0.029), patella (r = 0.223, *P* = 0.019), and calf (r = 0.222, *P* = 0.020). Postoperative joint pain scores showed a significant positive correlation with swelling in the calf (r = 0.224, *P* = 0.019). No significant correlations were found between age, cryotherapy duration, and postoperative inflammatory markers with swelling at various sites (*P* > 0.05), as shown in Table 5.

**Table 5:** Correlation analysis of swelling and factors [r (*P*-value)]

Variable	10 cm above patella	5 cm above patella	Patella	Calf	Ankle
Age	0.035 (0.719)	−0.002 (0.981)	0.002 (0.984)	0.041 (0.670)	0.091 (0.346)
BMI	0.153 (0.110)	0.208* (0.029)	0.223* (0.019)	0.222* (0.020)	0.038 (0.690)
Pre-op joint pain^a^	0.062 (0.522)	0.065 (0.502)	−0.012 (0.898)	−0.007 (0.943)	0.020 (0.836)
Post-op joint pain^a^	0.074 (0.440)	0.098 (0.310)	0.005 (0.960)	0.224* (0.019)	0.109 (0.256)
Post-op thigh pain^a^	0.017 (0.863)	0.048 (0.622)	−0.045 (0.641)	0.142 (0.140)	0.057 (0.557)
Cryotherapy duration	−0.035 (0.719)	−0.033 (0.733)	0.075 (0.433)	−0.019 (0.843)	0.022 (0.822)
Post-op WBC	0.023 (0.814)	−0.049 (0.613)	−0.058 (0.550)	−0.056 (0.563)	−0.030 (0.754)
Post-op CRP	0.061 (0.529)	−0.018 (0.855)	−0.021 (0.827)	0.005 (0.957)	−0.091 (0.343)

^a^ Pain was measured by VAS; * indicates statistical significance

### Multiple linear regression analysis

Multiple linear regression analysis showed that BMI is a key predictor for postoperative leg swelling, with a significant positive impact on all measurement sites except the ankle (*P* < 0.05). The effect size showed an anatomical gradient: 5 cm above patella (β = 0.238) > calf (β = 0.229) > patella (β = 0.191) > 10 cm above patella (β = 0.179), indicating that for every 1 unit increase in BMI, the proximal leg circumference increases by 0.18–0.24 cm. Pain indicators showed site-specific effects: swelling at the 10 cm above patella site was marginally affected by preoperative joint pain on touch (β = 0.184, *P* = 0.052), calf swelling was significantly influenced by postoperative joint pain at rest (β = 0.213, *P* = 0.022), and ankle swelling was only associated with postoperative thigh pain at rest (β = 0.228, *P* = 0.017). The overall explanatory power of the models was moderate (adjusted R^2^ = 2.7%–8.5%), with the 5 cm above patella (F = 4.937, *P* = 0.009) and calf (F = 6.041, *P* = 0.003) models showing the strongest significance. Methodological validation showed that all VIFs were < 1.10, and Bootstrap confirmed the robustness of the BMI effect (95% CI did not cross zero), although residual analysis suggested that the ankle model should be interpreted with caution (Table 6).

**Table 6:** Multiple linear regression analysis of lower limb swelling

Measurement site	Significant predictors	β Coefficient	*P*-Value	Adjusted R^2^	F Value	Model *P-*Value
**10 cm above patella**	BMI	0.179	0.058	0.055	4.164	0.018
	Pre-op joint pain on touch	0.184	0.052			
**5 cm above patella**	BMI	0.238	0.012	0.067	4.937	0.009
**Patella**	BMI	0.191	0.046	0.027	4.077	0.046
**Calf**	BMI	0.229	0.014	0.085	6.041	0.003
	Post-op joint pain at rest	0.213	0.022			
**Ankle**	Post-op thigh pain at rest	0.228	0.017	0.043	5.896	0.017

## Discussion

This study reveals the temporal variation patterns of swelling in 5 sites of the lower limbs in the early stage after TKA. Swelling was most prominent around the superior border of the patella, 5 cm, and 10 cm above the patella, with swelling significantly higher than the calf and ankle, with average dimensional differences of 3.62–3.76 cm and 0.08–1.89 cm, respectively (Table 2). This result aligns closely with the anatomical structure of the knee joint and the surgical trauma. The patella and suprapatellar areas are dense attachment zones for the quadriceps tendon, joint capsule, and synovium. Surgical procedures such as soft tissue dissection, osteotomy, and prosthesis implantation directly damage blood vessels, lead to tissue fluid exudation, and promote inflammatory cell infiltration in these areas. Ischemia–reperfusion injury caused by the tourniquet significantly activates local neutrophil infiltration and increases endothelial permeability, resulting in the accumulation of inflammatory exudates [11]. Regarding the temporal variation patterns, peak swelling occurred between postoperative days 3 and 4. Previous studies have shown that CRP, an acute-phase protein, peaks between postoperative days 1–3 [12, 13], with levels averaging 57.6 mg/L on day 4 [14], and returning to baseline levels within 2 weeks to 1 month after surgery [15]. Studies on Chinese TKA patients also showed a similar trend in CRP levels, with levels peaking on the third postoperative day at a concentration of 75.98 ± 40.01 mg/L [16, 17]. The peak swelling observed in our study between days 3 and 4 post-surgery corresponds to the reported peak of systemic inflammatory markers such as CRP. This temporal association supports the hypothesis that swelling may be a macroscopic manifestation of the local inflammatory response. This finding suggests that the 72-h postoperative period is crucial for inflammatory exudation and should be closely monitored for proximal swelling, with interventions prioritized during this phase.

Multiple regression analysis in this study identified BMI as a core predictor for swelling in most parts of the lower limb, except for the ankle, with a stronger impact on the proximal knee tissues (5 cm above the patella, calf, and patella) than on distal areas (10 cm above the patella). Obese patients (BMI ≥ 30 kg/m^2^) exhibited swelling levels 28.6–39.7% greater than those of normal BMI groups, with the largest differences observed at the 5 cm above the patella and calf sites (4.81 ± 3.34 cm vs. 3.45 ± 2.12 cm; 3.06 ± 3.44 cm vs. 1.17 ± 2.26 cm). This phenomenon may be related to the following mechanisms: from the perspective of metabolic inflammation, obese patients exhibit higher levels of serum tumor necrosis factor-alpha (TNF-α), interleukin (IL)−1β, and IL-6, which are produced by macrophages in adipose tissue and play a significant role in the pathogenesis of KOA. Elevated levels of these cytokines have been found in the synovial fluid, synovium, subchondral bone, and cartilage of KOA patients [18]. Although some studies suggest that TNF-α decreases after TKA, levels remain significantly higher in obese patients compared to normal BMI patients, with the chronic low-grade inflammatory state associated with obesity enhancing the vascular endothelial sensitivity to surgical trauma, thereby exacerbating the postoperative inflammatory response [19]. Due to similar mechanisms, obesity is also an important risk factor for lymphatic pain and arm swelling in breast cancer patients [20]. In terms of microcirculatory impairment, patients with high BMI may have concurrent microvascular damage, characterized by thickening of the vascular wall, narrowing of the lumen, and significant reductions in lymphatic and venous return efficiency. The failure of fluid to return promptly from the interstitial space further promotes the development of swelling [21]. A Chinese study indicates that obesity, sarcopenia, and sarcopenic obesity are highly prevalent among end-stage KOA patients [22]. Since sarcopenia is associated with elevated levels of IL-6, IL-17A, and TNF-α [23], patients with sarcopenic obesity may face a higher risk of swelling due to this combined inflammatory burden. However, this study did not address muscle mass in patients, as it requires more complex measurement techniques in clinical practice, which could be explored in future studies. Based on these findings, BMI ≥ 30 kg/m^2^ could serve as a marker for identifying patients at higher risk of pronounced swelling, who may benefit from targeted preventive interventions. Considering the difficulty of short-term weight loss in obese KOA patients, interventions targeting the control of pro-inflammatory cytokine levels could optimize postoperative swelling management.

From this study, there is no statistically significant differences were found in other demographic subgroups. Patients aged 65–75 accounted for 58.2% of the sample. Due to age-related declines in physical function, the inflammatory response patterns in the elderly tend to converge, which may obscure potential age-related differences. The study population consisted of 76.4% female patients, with swelling at all sites lower than in males, possibly due to the larger cross-sectional area of the male quadriceps, resulting in more severe muscle fiber rupture during surgery and increased inflammatory exudation during tissue repair. However, this difference did not reach statistical significance. Additionally, patients with underlying diabetes exhibited less swelling compared to non-diabetic patients, a finding that appears to contradict the theoretical assumption that diabetic individuals, being in a chronic low-grade inflammatory state, are more prone to swelling. A likely explanation for this discrepancy is that all diabetic patients, under our clinical protocol, underwent stringent preoperative glycemic optimization, which may have effectively controlled the chronic low-grade inflammation associated with diabetes. This suggests that effective preoperative management may alleviate diabetes-related microvascular dysfunction and chronic inflammation, thereby normalizing their acute postoperative inflammatory response and swelling risk to levels comparable to those of non-diabetic patients. These findings underscore the critical importance of comprehensive comorbidity management prior to surgery and merit further exploration in future studies.

A comprehensive analysis of the factors contributing to swelling at various sites and the regression model results, in combination with the relationship between inflammatory markers and relevant factors, reveals differences in the mechanisms underlying postoperative lower limb swelling at different sites, highlighting the unique driving factors for swelling in the proximal knee (suprapatellar regions), calf, and ankle. In the proximal areas (10 cm/5 cm above patella and patella), the multiple linear regression model shows a gradient effect of BMI as a predictor, with the 5 cm above patella area being most affected (β = 0.238), followed by the patella. This pattern suggests that, in addition to the pro-inflammatory effect of adipose tissue, obesity may also obstruct fluid return through compression of superficial lymphatic vessels and veins, exacerbating swelling. Furthermore, preoperative joint pain on touch was associated with more significant swelling at the 10 cm above patella (*P* = 0.028), and patients with preoperative pain during activity had significantly higher WBC counts (11.04 vs 9.79 × 10⁹/L, *P* = 0.012), suggesting that peripheral nerve sensitization in the pain area could contribute to increased capillary permeability and potentially exacerbate local microcirculatory impairment, which might in turn promote swelling. Some studies have reported that increased pain sensitivity is associated with higher postoperative pain intensity and poorer patient-reported outcomes after TKA [24]. This study’s findings suggest that a similar conclusion may hold for postoperative swelling. Shao et al. [25] found that a nomogram constructed based on BMI, KOA duration, surgical duration, and intraoperative blood loss could predict postoperative swelling. The disease duration of KOA is related to the preoperative joint pain observed in this study. KOA pain is maintained by complex peripheral and central nervous system mechanisms. Tissue damage and inflammatory responses are the driving factors of pain, and the sensitization of peripheral nociceptors and spinal cord neurons is an important condition for pain conduction and maintenance [26]. Some studies have shown that the manifestations of pain symptoms can better predict the severity of patients undergoing TKA compared with the Kellgren–Lawrence grade [27], and patients with KOA have a longer course of widespread pain before surgery [28]. Therefore, the prediction of swelling by the timing of preoperative knee pain proposed in this study may be more specific and easier to obtain compared with the duration of the course of KOA. In conclusion, obese patients with preoperative joint tenderness may have an elevated risk for proximal swelling and require key screening and early intervention.

The calf swelling model showed the highest explanatory power (adjusted R^2^ = 0.085), and in addition to the pro-inflammatory-mechanical effect of BMI, the study found an independent contribution of joint pain at rest to calf swelling (β = 0.213, *P* = 0.022). This finding corroborates the analysis of inflammatory markers, as patients with joint rest pain had significantly elevated CRP levels (32.2 vs 21.3 mg/L, *P* = 0.035), indicating that inflammation related to pain may exacerbate tissue exudation by altering endothelial function. This mechanism suggests that intervention strategies for calf swelling should be synergistic, focusing on joint pain at rest during pain assessments and implementing various measures to inhibit the release of inflammatory cytokines, improve local circulation, and modulate pain transmission.

The ankle swelling regression model showed a significant association with thigh rest pain (β = 0.228, *P* = 0.017), but no statistical correlation with other risk factors, suggesting that ankle swelling may be caused by changes in sympathetic nerve tension due to pain, leading to abnormal vasomotor function and altered fluid distribution at the distal site. However, the explanatory power of the ankle swelling model was relatively low (adjusted R^2^ = 0.043), and unmeasured neurophysiological factors may be involved, warranting further investigation.

This study did not identify a clear association between cryotherapy and swelling, but this finding should not be interpreted as evidence against the efficacy of cryotherapy. In this study, daily cryotherapy duration was recorded, but it did not control for factors such as temperature, intervals between sessions, or single-session duration. Studies have shown that there is relatively clear evidence for the effect of cryotherapy on postoperative pain, while the effect on swelling still needs to be explored [29]. Various cryotherapy parameters can affect its swelling control effectiveness [10]. Subgroup analysis indicated that the group receiving cryotherapy ≥ 60 min/day had higher swelling at the superior patella (4.55 ± 2.74 cm) than those receiving shorter durations, possibly due to longer cryotherapy being administered to patients with more severe swelling, but without achieving significant swelling control effects. The rationale behind this is that cryotherapy primarily inhibits vascular permeability early after surgery; if swelling develops and cryotherapy duration is extended thereafter, the critical window for inhibiting exudation may be missed, weakening its effectiveness [10]. Therefore, the heterogeneity of cryotherapy parameters may be one reason why no significant correlation was found between cryotherapy duration and swelling. Further studies should clarify the mechanisms of different temperatures in cryotherapy and optimize cryotherapy protocols.

This study has several limitations. First, in terms of sampling methodology, the use of convenience sampling rather than random sampling may introduce selection bias. Nevertheless, we consecutively included all patients who met the criteria to ensure the representativeness of the sample as much as possible. Second, there are inherent challenges in data collection. To minimize patient burden and pain, inflammatory markers were measured only once within 24 h postoperatively based on routine clinical practice, thereby preventing an analysis of the full dynamic relationship between CRP and swelling and likely missing associations during the critical period of peak inflammation. Similarly, for cryotherapy, only the patient-reported duration was recorded. Some parameters, such as temperature and pressure, were not captured due to a lack of standardization in clinical practice and inherent measurement challenges, reflecting a common bottleneck in this field of research. Additionally, although all surgeries followed highly standardized procedures (e.g., uniform use of tourniquets and tranexamic acid protocols), which minimized inter-individual variations in intraoperative variables, we cannot completely rule out potential biases caused by certain personalized factors, such as individual differences in soft tissue handling and analgesic treatments. Meanwhile, variables like tourniquet duration, pressure, and surgical duration exhibited low variability under the standardized procedures and were thus not included in the analysis. However, their potential confounding effects in broader surgical scenarios still require investigation in future studies. Finally, the explanatory power (R^2^ values) of some statistical models was limited. This indicates that although identified factors such as obesity and pain characteristics are significant risk indicators, they only account for a part of the complex mechanisms leading to swelling. We have verified the robustness of the model coefficients through methods such as the Bootstrap method, but the interpretation of the results should be conducted with caution. In future higher-quality study designs, multi-center studies, random sampling, continuous monitoring of inflammatory markers, standardized recording of cryotherapy parameters, and the inclusion of a wider range of potential predictors could be adopted to construct a more comprehensive prediction model for swelling.

## Conclusion

In summary, this study identified BMI and preoperative pain as statistically significant predictors of proximal joint swelling in the first 3 to 4 days after TKA. However, the low explanatory power of our models indicates that these factors account for only a small portion of the swelling variability, and unmeasured factors likely play a more substantial role. Therefore, obese KOA patients with preoperative joint tenderness may have an elevated risk, but this should be interpreted with caution. Future research should focus on identifying these other factors, along with dynamic inflammation monitoring and cryotherapy optimization, to build a more comprehensive predictive model.

## Data Availability

The data that support the findings of this study are available from the corresponding author upon reasonable request.
